# Bacterial infections epidemiology and factors associated with multidrug resistance in the northern region of Ghana

**DOI:** 10.1038/s41598-022-26547-7

**Published:** 2022-12-21

**Authors:** Jean-Pierre Gnimatin, Enoch Weikem Weyori, Shimea M. Agossou, Martin Nyaaba Adokiya

**Affiliations:** 1 Department of Social and Behavioral Change, School of Public Health, University for Development Studies, Tamale, Ghana; 2 Zonal Public Health Reference Laboratory, Tamale, Ghana; 3 Department of Epidemiology, Regional Institute of Public Health, University of Abomey-Calavi, Ouidah, Benin; 4 Department of Epidemiology, Biostatistics and Disease Control, School of Public Health, University for Development Studies, Tamale, Ghana

**Keywords:** Antimicrobials, Bacteria, Risk factors, Epidemiology

## Abstract

Bacterial infections caused by multidrug resistant organisms are a major global threat. There is still a knowledge gap on this situation in the Northern Region of Ghana. This study determined the prevalence and resistance profile of bacterial infections. It also identified factors associated with multidrug resistance in the study area. This was a retrospective cross-sectional design and it analyzed data from the samples received at the Tamale Zonal Public Health Reference Laboratory from June 2018 to May 2022. The data were analyzed using the R software version 4.2.0. Univariate and multivariable binary logistic regression analyses were used to determine the factors associated with multidrug resistance. The samples included all specimen types possible. The specimens were collected for the purpose of clinical bacteriology diagnostics. Overall a total of 1222 isolates were obtained. The three (3) main bacteria responsible for infections were: *Klebsiella* spp*.* (27%), *Moraxella* spp*.* (22%), *Escherichia* spp*.* (16%). High resistance levels were found against the tested antibiotics and about 41.60% of the bacterial strains isolated were multidrug resistant. Hospitalization was associated with multidrug resistance in univariate (COR 1.96; 95% CI 1.43–2.71; P-value < 0.001) and multivariable analyses (AOR 1.78; 95% CI 1.28–2.49; P-value < 0.001). There is the need for further research on the molecular epidemiology of antibiotic resistance genes in the study area to effectively control the spread of multidrug resistant pathogens. In addition, efforts to build the capacity of health professionals on infection prevention and control as well as diagnostic and antimicrobial stewardship needs urgent attention.

## Introduction

Antimicrobial resistance (AMR) is a global threat. Infections caused by multidrug resistant organisms (MDROs), which result in significantly fewer treatment options, are ranked among the top global public health concerns^1,2^. Antibiotics are widely prescribed and used for the treatment of patients in hospital facilities. In these settings, bacteria are more confronted with antibiotic and these organisms must adapt to resist the effect of antibiotics to survive. Therefore, the bacteria develop acquired resistance to one or more of these antibiotics^3^. This has led to the emergence of strains called Multidrug Resistance (MDR) which can withstand the action of at least one antibiotic agent from three or more antibacterial categories^4^. These resistant infectious agents frequently cause infections in community and hospital settings. They can be involved in any type of infection, including respiratory, urinary tract, bloodstream (sepsis), post-surgical (wound) and pneumonia infections^5–7^. Several studies in many African countries including Ghana have revealed high rates of multidrug resistant pathogens in hospital settings^8–15^. In Ghana, a 2015 national laboratory surveillance reported high levels of pathogen resistant to most antimicrobials in the country^16^. In addition, the results of a study on antibiotic prescription at the Tamale Teaching Hospital (TTH), which is the only tertiary level referral hospital in the North of Ghana, revealed a high proportion of antibiotic abuse coupled with a high prevalence of incomplete treatment, off-label prescriptions and potential interactions. About 385 examples of various antibiotic misuse were discovered, including 335 prescription errors and 50 unfinished treatments with the most prevalent prescription error being on treatment length (29.6%)^17^. However, limited knowledge is available at the regional level regarding the prevalence of bacterial pathogens responsible for infections and the factors associated with their multidrug resistance. Therefore, the need to investigate infections caused by multidrug resistant bacteria in this region by analyzing recent available data and to provide useful information to stakeholders for guiding decision-making and control programs implementation. Thus, we hypothesized that there is an association between infections with multidrug resistant pathogens and risk factors such as sex, age group, hospitalization status and causative bacteria. This study, therefore, determined the prevalence and resistance profile of bacteria responsible for infections in northern Ghana. It also determined the factors associated with infections caused by multidrug resistant strains.

## Methods

### Study setting and design

**Figure 1:**
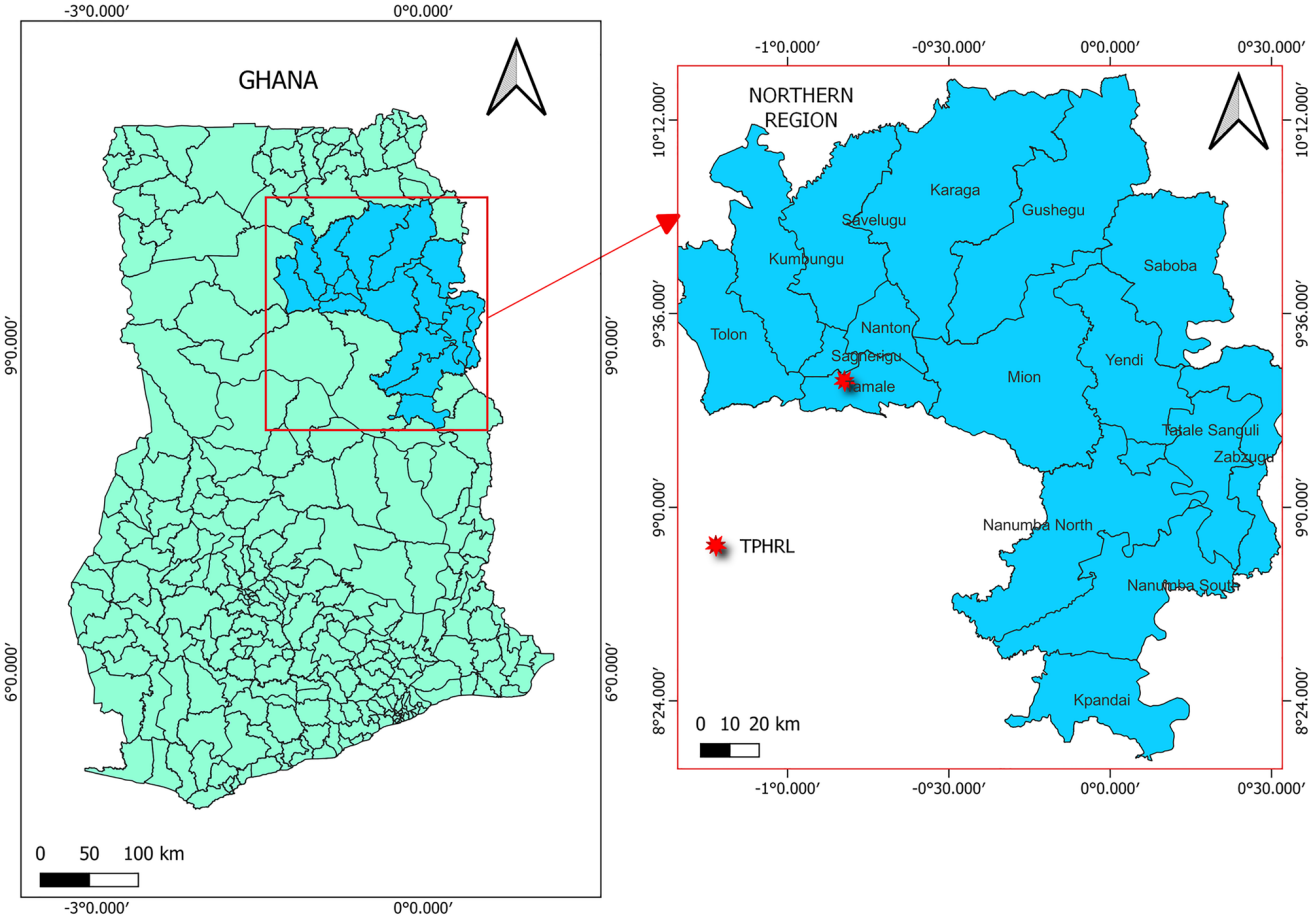
Geographical map of the study area.

The data was collected from the Tamale Zonal Public Health Reference Laboratory (TZPHRL). It is one of the three Zonal Public Health Reference Laboratories in Ghana. The TZPHRL is located in Tamale, the capital town of the Northern Region of Ghana (Fig. 1)*.* This figure shows the geographical area of the study setting. This was a retrospective cross-sectional study. It analysed clinically recorded data from the samples received for bacterial culture and antibiotic susceptibility testing at the TZPHRL over a period of 48 months or four years (Year 1: June 2018–May 2019, Year 2: June 2019–May 2020, Year 3: June 2020–May 2021, Year 4: June 2021–May 2022).

### Pre-study realization: bacteria isolation, identification, and antibiotic susceptibility testing

The TZPHL performed routine microbiological investigations on all clinical samples received using standard operating procedures to isolate bacteria responsible for infections. Bacterial isolates were then identified using Gram morphology, routine biochemical tests (catalase testing, oxidase testing, urease and substrate utilization tests), and in some instances the API 20E system (bioMérieux SA, Marcy l’Etoile, France). Susceptibility tests were performed by the disk diffusion method^18^. In addition, inhibition zone sizes were measured and reported in millimeters according to the guidelines of the Clinical and Laboratory Standards Institute (CLSI)^19^. Specific gram-negative and gram-positive antimicrobial disks were selected for gram-negative and gram-positive isolates. The disks tested and their concentrations in micrograms included: Amikacin (30), Amoxicillin (30), Azithromycin (15), Cefotaxime (30), Cefoxitin (30), Ceftazidime (30), Ceftriaxone (30), Chloramphenicol (30), Ciprofloxacin (05), Erythromycin (15), Gentamicin (10), Meropenem (10), Penicillin V (10), Tetracycline (30), and Trimethoprim/Sulfamethoxazole (1.2).

Once the bacterial culture, isolation, identification and antibiotic susceptibility tests were done and the results were entered into the Microbiology electronic database of the Laboratory. The entered data were verified by the Chief laboratory technician and the Chief data manager of the TZPHRL.

### Sampling and data extraction

There was no predefined or calculated sample size for this study. The study included all samples of suspected bacterial infections received at the TZPHRL for microbiological testing including bacterial culture, isolation, identification and susceptibility testing received from June 2018 to May 2022. For data extraction, the Microbiology electronic records of the TZPHRL were used. Apart from the records with no bacterial growth, information about all cultured samples of patients such as age, sex, specimen type, hospitalization status, bacterial isolates, year of isolation and antibiotic susceptibility patterns were collected. In total, 1222 positive bacterial culture were recorded over the study period.

### Inclusion and exclusion criteria

This study included clinical records from patients whose samples have been received for bacterial culture and antibiotic susceptibility testing at the Microbiology section of the TZPHRL from June 2018 to May 2022. Records from patients whose samples culture resulted in no bacterial growth and records on fungi elements were excluded.

### Definitions

From the antimicrobial susceptibility tests, different resistance patterns are observed for each bacterial strain. When an antibiotic agent had the expected inhibition effect on a bacterium, it is said to be sensitive or susceptible to that antibiotic agent. A bacterium is identified as resistant to a given antibiotic agent when it thwarts the effect that is supposed to kill the organism and continues to grow in its presence. Bacteria that are resistant to at least one antibiotic agent in any three or more antibacterial categories are identified as multidrug resistant isolates^4^.

### Study variables

The dependent variable was the multidrug resistance profile among patients screened for bacterial infections at the TZPHRL. The independent variables were the sex of the patient, age of the patient, age group, specimen type, bacteria isolated and hospitalization status of the patient (In-patient/Out-patient).

### Statistical analysis

The data extraction and analysis were conducted between June and August 2022. The data was extracted, cleaned and made ready for analysis using Microsoft Excel 2019. The formal analysis was conducted using the R software version 4.2.0. The age of the patients was presented as descriptive statistics such as mean, median, range, and quantiles. The distribution of age has been tested using the Shapiro–Wilk normality test. The gtsummary^20^ package version 1.6.1 was used to create publication-ready analytical and summary tables where comparisons were done for continuous and categorical data. The Kruskal–Wallis rank-sum test was used to compare non-normally distributed quantitative variables. The Pearson Chi-squared test was used to compare categorical data. Univariate and multivariable binary logistic regression analyses were used to determine the factors associated with multidrug resistant (MDR) infections. Cases with missing values were excluded from the regression analyses. All variables associated with MDR at a P-value < 0.200 in the univariate model were included in the initial multivariable model. The “stepAIC” function was then applied to the initial multivariable model using a stepwise method with both forward and backward selection to get the final multivariable logistic regression model. The results were presented as odds ratios (ORs) with 95% confidence intervals (CIs) and all analyses were conducted with a P-value of 0.05 considered statistically significant.

### Ethical approval

This study was approved by the Committee of Human Research and Publication Ethics of the Kwame Nkrumah University of Science and Technology (KNUST) (Ref: CHRPE/AR/060/22; Date 15/02/2022). The protocol amendment and a waiver of informed consent have been approved by the same Committee (Ref: CHRPE/AP/163/22; Date 05/05/2022). We also received permission from the Tamale Regional Health Directorate. All methods were carried out in accordance with relevant ethical guidelines and regulations.

## Results

### Patients’ characteristics

Table 1 presents the baseline characteristics of the patients recorded from June 2018 to May 2022 at Tamale Zonal Public Health Reference Laboratory (TZPHRL). A total of 1222 positive bacterial cultures including 593 (48.5%) females were recorded. The age of the patients of the samples received ranged from 0 to 105 years and the median age was 41 years (28, 60). During the four (4) years covered by this study, there was no statistically significant difference in the number of samples received per year by sex of patients (P-value = 0.300). In the first, second, third and fourth years, 149 (55.0%), 150 (50.0%), 147 (49.0%) and 147 (47.0%) of the samples were from females respectively. A statistically significant difference was observed for the distribution of age groups over the years (P-value = 0.010). The majority of samples were collected from patients in the 24–44 age group (39.0%) and 60 + age group (28%). Patients under 5 years of age represented only 2.40% of the samples. On the patient's hospitalization status, we found that 81.0% of the samples were from hospitalized patients.

**Table 1:** Baseline characteristics of patients recorded during the study period.

Characteristic	Year 1 N = 284^1^	Year 2 N = 309^1^	Year 3 N = 308^1^	Year 4 N = 321^1^	Overall N = 1222^1^	p-value^2^
**Sex**						0.300
Female	149 (55%)	150 (50%)	147 (49%)	147 (47%)	593 (50%)	
Male	123 (45%)	149 (50%)	151 (51%)	167 (53%)	590 (50%)	
**Median age**	39 (27, 60)	41 (28, 58)	42 (29, 60)	42 (28, 60)	41 (28, 60)	0.800
**Age group**						0.010
Under 5	14 (5.4%)	8 (2.8%)	4 (1.3%)	1 (0.3%)	27 (2.4%)	
5–14	15 (5.8%)	9 (3.1%)	17 (5.7%)	8 (2.6%)	49 (4.3%)	
15–24	16 (6.2%)	23 (8.0%)	28 (9.4%)	30 (9.9%)	97 (8.5%)	
25–44	102 (40%)	120 (42%)	106 (36%)	114 (38%)	442 (39%)	
45–59	39 (15%)	54 (19%)	55 (19%)	63 (21%)	211 (18%)	
60 +	69 (28%)	66 (25%)	78 (29%)	79 (29%)	292 (28%)	
**Hospitalization status**						0.200
Inpatient	236 (83%)	258 (83%)	238 (77%)	255 (79%)	987 (81%)	
Outpatient	48 (17%)	51 (17%)	70 (23%)	66 (21%)	235 (19%)	

^1^n (%); Median (IQR); ^2^Pearson's Chi-squared test; Kruskal–Wallis rank sum test.

### Biological samples

Supplementary Table S1 online shows the samples received at the TZPHRL between June 2018 and May 2022. We found that sputum was the most common sample with a percentage of 68.0%, followed by urine (11.0%), high vaginal swab (6.4%), wound swab (6.1%) and blood (5.1%).

### Epidemiology of bacterial infections

Table 2 shows the common bacterial genus. The results revealed the five (5) main bacterial genus responsible for the infections isolated from the TZPHRL. These were: *Klebsiella* spp*.* (27.0%), *Moraxella* spp*.* (22.0%), *Escherichia* spp*.* (16.0%), *Pseudomonas* spp*.* (13.0%), and *Staphylococcus* spp*.* (7.7%). *Sphingomonas* spp., *Shewanella* spp., *Providencia* spp., *Photobacterium* spp*.* and *Gardnerella* spp*.* were rarely isolated (n = 1) during the study period. Each of these bacterial genus had a prevalence of less than 0.1% in total.

**Table 2:** Bacteria isolated from the TZPHRL during the study period.

Isolated bacteria	Year 1 N = 284^1^	Year 2 N = 309^1^	Year 3 N = 308^1^	Year 4 N = 321^1^	Overall N = 1222^1^	p-value^2^
						< 0.001
*Klebsiella* spp*.*	50 (18%)	151 (49%)	116 (38%)	17 (5.3%)	334 (27%)	
*Moraxella* spp*.*	4 (1.4%)	52 (17%)	113 (37%)	95 (30%)	264 (22%)	
*Escherichia* spp*.*	107 (38%)	77 (25%)	16 (5.2%)	1 (0.3%)	201 (16%)	
*Pseudomonas* spp*.*	4 (1.4%)	7 (2.3%)	39 (13%)	104 (32%)	154 (13%)	
*Staphylococcus* spp*.*	5 (1.8%)	8 (2.6%)	10 (3.2%)	71 (22%)	94 (7.7%)	
*Enterobacter* spp*.*	56 (20%)	9 (2.9%)	1 (0.3%)	1 (0.3%)	67 (5.5%)	
*Acinetobacter* spp*.*	46 (16%)	2 (0.6%)	1 (0.3%)	0 (0%)	49 (4.0%)	
*Proteus* spp*.*	1 (0.4%)	0 (0%)	8 (2.6%)	5 (1.6%)	14 (1.1%)	
*Raoultella* spp*.*	1 (0.4%)	0 (0%)	1 (0.3%)	9 (2.8%)	11 (0.9%)	
*Streptococcus* spp*.*	0 (0%)	2 (0.6%)	1 (0.3%)	6 (1.9%)	9 (0.7%)	
*Salmonella* spp*.*	2 (0.7%)	0 (0%)	1 (0.3%)	2 (0.6%)	5 (0.4%)	
*Serratia* spp*.*	0 (0%)	0 (0%)	0 (0%)	4 (1.2%)	4 (0.3%)	
*Corynebacterium* spp*.*	3 (1.1%)	0 (0%)	0 (0%)	0 (0%)	3 (0.2%)	
*Citrobacter* spp*.*	2 (0.7%)	0 (0%)	0 (0%)	0 (0%)	2 (0.2%)	
*Enterococcus* spp*.*	2 (0.7%)	0 (0%)	0 (0%)	0 (0%)	2 (0.2%)	
*Micrococcus* spp*.*	0 (0%)	0 (0%)	1 (0.3%)	1 (0.3%)	2 (0.2%)	
*Pantoea* spp*.*	0 (0%)	0 (0%)	0 (0%)	2 (0.6%)	2 (0.2%)	
*Gardnerella* spp*.*	1 (0.4%)	0 (0%)	0 (0%)	0 (0%)	1 (< 0.1%)	
*Photobacterium* spp*.*	0 (0%)	0 (0%)	0 (0%)	1 (0.3%)	1 (< 0.1%)	
*Providencia* spp*.*	0 (0%)	1 (0.3%)	0 (0%)	0 (0%)	1 (< 0.1%)	
*Shewanella* spp*.*	0 (0%)	0 (0%)	0 (0%)	1 (0.3%)	1 (< 0.1%)	
*Sphingomonas* spp*.*	0 (0%)	0 (0%)	0 (0%)	1 (0.3%)	1 (< 0.1%)	

^1^n (%); ^2^Pearson's Chi-squared test.

Supplementary Table S2 online shows the sex distribution of bacteria isolated at the TZPHRL. Bacteria such as *Klebsiella* spp*.*, *Escherichia* spp., *Staphylococcus* spp*.* and *Enterobacter* spp*.* were more common in women than men, with percentages of 51.0%, 62.0%, 56.0% and 53.0%, respectively. In contrast, bacteria such as *Moraxella* spp*.* (56.0%) *Pseudomonas* spp*.* (59.0%) and *Acinetobacter* spp*.* (54.0%), were isolated more frequently in men than women.

Supplementary Table S3 online presents the distribution of bacteria isolated according to age group. *Staphylococcus* spp*.* was the most common bacterium among patients under-5 age group, accounting for 48%. *Moraxella* spp*.* was the most common bacterial genus (22.0%) in the 5–14 age group. The two most isolated bacterial genus in the 45–59 age group were *Klebsiella* spp*.* and *Moraxella* spp*.* (each accounting for 26.0%). Among people aged 15–24, *Escherichia* spp*.* was the most isolated bacteria (29.0%). *Klebsiella* spp*.* was the most commonly isolated bacteria in the 25–44 and 60 + age groups, accounting for 29.0% and 30.0% respectively.

### Antibiotic resistance profile of the isolated bacteria

In general, high rates of resistance were recorded against the different antibiotics tested. As it is shown in Table 3, the highest resistance rates were found with Penicillin V. against which 95.2% (n = 40) of the tested bacteria showed non-sensitivity. It is followed by Amoxicillin against which 77.4% of the tested bacteria were resistant. The other antibiotics with a high rate of resistance were: Cefoxitin (74.4%), Tetracycline (71.3%), Trimethoprim/Sulfamethoxazole (68.2%), Ceftriaxone (66.7%), Cefotaxime (62.4%), Chloramphenicol (54.8%) and Erythromycin (51.3%). Low rates of resistance were recorded against other antibiotics such as Amikacin (15.4%), Meropenem (24.0%), Gentamicin (30.6%), and Ceftazidime (38.5%).

**Table 3:** Antibiotic resistance profile of bacteria isolated at the TZPHRL.

	AMK	AMX	PNV	AZM	ERY	CHL	CIP	CAZ	CRO	CTX	FOX	GEN	MEM	SXT	TCY
*Klebsiella* spp*.*	14 (5.10%)	223 (74%)	–	128 (42%)	–	2 (50%)	118 (42%)	143 (46%)	118 (64%)	91 (63%)	3 (100%)	69 (33%)	11 (22%)	77 (68%)	5 (71%)
*Moraxella* spp*.*	92 (38%)	2 (29%)	–	4 (36%)	–	–	152 (69%)	70 (28%)	1 (100%)	2 (67%)	–	50 (28%)	67 (27%)	2 (67%)	1 (50%)
*Escherichia* spp*.*	12 (7.40%)	147 (81%)	–	88 (48%)	–	0 (0%)	82 (52%)	90 (48%)	78 (63%)	70 (60%)	–	53 (38%)	4 (18%)	35 (76%)	5 (71%)
*Pseudomonas* spp*.*	17 (12%)	20 (87%)	–	13 (57%)	–	–	29 (21%)	33 (23%)	14 (93%)	14 (93%)	–	26 (21%)	26 (21%)	6 (75%)	1 (100%)
*Enterobacter* spp*.*	4 (7.50%)	51 (84%)	–	22 (35%)	–	2 (67%)	29 (48%)	25 (42%)	23 (68%)	21 (64%)	–	14 (36%)	1 (20%)	15 (68%)	2 (100%)
*Acinetobacter* spp*.*	5 (12%)	39 (87%)	–	11 (24%)	–	–	15 (35%)	23 (51%)	22 (88%)	18 (90%)	–	13 (37%)	1 (20%)	10 (67%)	0 (0%)
*Proteus* spp*.*	1 (10%)	4 (40%)	–	9 (82%)	–	2 (100%)	3 (25%)	3 (30%)	4 (57%)	1 (17%)	–	2 (25%)	0 (0%)	4 (80%)	–
*Raoultella* spp*.*	0 (0%)	7 (78%)	–	1 (11%)	–	–	5 (83%)	3 (33%)	6 (67%)	4 (50%)	–	1 (17%)	–	4 (100%)	–
*Salmonella* spp*.*	0 (0%)	4 (100%)	–	0 (0%)	–	–	2 (50%)	2 (40%)	–	0 (0%)	–	0 (0%)	–	4 (100%)	–
*Serratia* spp*.*	0 (0%)	4 (100%)	–	1 (25%)	–	–	1 (25%)	1 (25%)	1 (33%)	0 (0%)	–	0 (0%)	–	–	–
*Staphylococcus* spp*.*	2 (67%)	1 (100%)	40 (95%)	33 (42%)	33 (49%)	31 (52%)	23 (42%)	0 (0%)	1 (100%)	2 (67%)	27 (71%)	19 (36%)	0 (0%)	31 (55.36%)	51 (70%)
*Citrobacter* spp*.*	0 (0%)	1 (50%)	–	0 (0%)	–	–	0 (0%)	0 (0%)	–	–	–	0 (0%)	–	–	–
*Pantoea* spp*.*	0 (0%)	1 (50%)	–	1 (50%)	–	–	2 (100%)	2 (100%)	2 (100%)	1 (100%)	1 (100%)	0 (0%)	–	1 (100%)	–
*Photobacterium* spp*.*	0 (0%)	0 (0%)	–	1 (100%)	–	–	1 (100%)	0 (0%)	1 (100%)	0 (0%)	–	0 (0%)	–	–	–
*Providencia* spp*.*	0 (0%)	1 (100%)	–	1 (100%)	–	–	0 (0%)	0 (0%)	–	–	–	0 (0%)	–	–	–
*Shewanella* spp*.*	0 (0%)	–	–	–	–	–	–	1 (100%)	–	–	–	0 (0%)	1 (100%)	–	–
*Sphingomonas* spp*.*	0 (0%)	1 (100%)	–	1 (100%)	–	–	0 (0%)	0 (0%)	1 (100%)	1 (100%)	–	–	–	1 (100%)	–
*Streptococcus* spp*.*	–	–	–	5 (71%)	5 (83%)	5 (62%)	0 (0%)	–	–	1 (14%)	–	1 (100%)	–	–	5 (71%)
*Corynebacterium* spp*.*	–	–	–	0 (0%)	1 (33%)	1 (50%)	0 (0%)	–	–	–	1 (100%)	0 (0%)	–	0 (0%)	3 (100%)
*Enterococcus* spp*.*	–	–	–	2 (100%)	–	2 (100%)	1 (50%)	–	–	1 (100%)	–	0 (0%)	–	1 (100%)	2 (100%)
*Micrococcus* spp*.*	–	–	–	2 (100%)	–	1 (100%)	1 (50%)	–	–	–	–	0 (0%)	–	2 (100%)	1 (50%)
*Gardnerella* spp*.*	–	–	–	0 (0%)	–	0 (0%)	1 (100%)	–	–	–	–	1 (100%)	–	–	1 (100%)
Total	147/956 (15.40%)	506/654 (77.37%)	40/42 (95.24%)	323/758 (42.61%)	39/76 (51.32%)	46/84 (54.76%)	465/995 (46.73%)	396/1029 (38.48%)	272/408 (66.67%)	227/364 (62.36%)	32/43 (74.42%)	249/815 (30.55%)	111/462 (24.03%)	193/283 (68.20%)	77/108 (71.30%)

*AMK *Amikacin, *AMX* Amoxicillin, *AZM* Azithromycin, *CAZ* Ceftazidime, *CHL* Chloramphenicol, *CIP* Ciprofloxacin, *CRO* Ceftriaxone, *CTX* Cefotaxime, *ERY* Erythromycin, *FOX* Cefoxitin, *GEN* Gentamicin, *MEM* Meropenem, *PNV* Penicillin V, *SXT* Trimethoprim/Sulfamethoxazole, *TCY* Tetracyclin.

Table 3 shows the bacterial resistance profile for the tested antibiotics at TZPHRL between June 2018 and May 2022. *Klebsiella* spp. strains showed high resistance to Amoxicillin (74.0%), Tetracycline (71.0%), Trimethoprim/Sulfamethoxazole (68.0%), Ceftriaxone (64.0%) and Cefotaxime (63.0%). *Klebsiella* spp*.* strains showed high sensitivity to Amikacin with only 5.1% of resistance. The highest rate of resistance among *Moraxella* spp*.* strains were observed against Ciprofloxacin (69.0%). Strains of *Escherichia* spp., *Pseudomonas* spp., *Enterobacter* spp. and *Acinetobacter* spp. were highly resistant to Amoxicillin (81.0%, 87.0%, 84.0%, and 87.0% respectively) and to Ceftriaxone with resistance rates of 63.0%, 93.0%, 68.0%, and 88.0% respectively. These same bacteria showed resistance levels of 60.0%, 93.0%, 64.0%, and 90.0% to Cefotaxime, respectively. It was found that the strains of *Staphylococcus* spp*.* showed the highest levels of resistance to Penicillin V (95.0%), Tetracycline (70.0%) and Cefoxitin (71.0%).

### Prevalence of the multidrug resistance

The results show a high prevalence of multidrug resistant bacteria. In general, 41.6% of the bacteria strains isolated from the TZPHRL were multidrug resistant. Among the females, 45.0% were infected by multidrug resistant organisms (MDRO) compared to 38.0% among males. From Table 4, which shows the bivariate distribution of patient characteristics according to their multidrug resistance status, it revealed that 45.0% of hospitalized patients were infected with multidrug resistant bacteria compared to 28.0% of non-hospitalized patients. We also found that patients among the under-5 age group had a high prevalence of infection with multidrug resistance (56.0%). Among the most prevalent isolated bacteria, the highest rates of multidrug resistance were found among *Staphylococcus* spp*.* (56.0%), *Escherichia* spp*.* (56.0%) and *Enterobacter* spp*.* (54.0%) and those with the lowest multidrug resistance rates were *Pseudomonas* spp*.* (19.0%) and *Moraxella* spp*.* (25.0%).

**Table 4:** Bivariate distribution of patient characteristics according to their multidrug resistance status.

Characteristic	MDR− N = 714^1^ (58.40%)	MDR+ N = 508^1^ (41.60%)	Overall N = 1222^1^ (100%)	p-value^2^
**Sex**				0.019
Female	328 (55%)	265 (45%)	593 (100%)	
Male	366 (62%)	224 (38%)	590 (100%)	
**Age**	40 (28,58)	41 (28,62)	41 (28,60)	0.300
**Age group**				0.031
Under 5	12 (44%)	15 (56%)	27 (100%)	
5–14	33 (67%)	16 (33%)	49 (100%)	
15–24	49 (51%)	48 (49%)	97 (100%)	
25–44	280 (63%)	162 (37%)	442 (100%)	
45–59	128 (61%)	83 (39%)	211 (100%)	
60 +	176 (55%)	143 (45%)	319 (100%)	
**Hospitalization status**				< 0.001
Inpatient	544 (55%)	443 (45%)	987 (100%)	
Outpatient	170 (72%)	65 (28%)	235 (100%)	
**Isolated bacteria**				
*Acinetobacter* spp*.*	27 (55%)	22 (45%)	49 (100%)	
*Citrobacter* spp*.*	2 (100%)	0 (0%)	2 (100%)	
*Corynebacterium* spp*.*	2 (67%)	1 (33%)	3 (100%)	
*Enterococcus* spp*.*	0 (0%)	2 (100%)	2 (100%)	
*Enterobacter* spp*.*	31 (46%)	36 (54%)	67 (100%)	
*Escherichia* spp*.*	89 (44%)	112 (56%)	201 (100%)	
*Gardnerella* spp*.*	0 (0%)	1 (100%)	1 (100%)	
*Klebsiella* spp*.*	171 (51%)	163 (49%)	334 (100%)	
*Micrococcus* spp*.*	0 (0%)	2 (100%)	2 (100%)	
*Moraxella* spp*.*	198 (75%)	66 (25%)	264 (100%)	
*Pantoea* spp*.*	1 (50%)	1 (50%)	2 (100%)	
*Photobacterium* spp*.*	0 (0%)	1 (100%)	1 (100%)	
*Proteus* spp*.*	8 (57%)	6 (43%)	14 (100%)	
*Providencia* spp*.*	1 (100%)	0 (0%)	1 (100%)	
*Pseudomonas* spp*.*	125 (81%)	29 (19%)	154 (100%)	
*Raoultella* spp*.*	6 (55%)	5 (45%)	11 (100%)	
*Salmonella* spp*.*	3 (60%)	2 (40%)	5 (100%)	
*Serratia* spp*.*	3 (75%)	1 (25%)	4 (100%)	
*Shewanella* spp*.*	1 (100%)	0 (0%)	1 (100%)	
*Sphingomonas* spp*.*	0 (0%)	1 (100%)	1 (100%)	
*Staphylococcus* spp*.*	41 (44%)	53 (56%)	94 (100%)	
*Streptococcus* spp*.*	5 (56%)	4 (44%)	9 (100%)	

^1^n (%); Median (IQR); ^2^Pearson's Chi-squared test; Kruskal–Wallis rank sum test.

### Factors associated with the multidrug resistance

Tables 5 and 6 show the univariate and the multivariable logistic regression analyses of the risk factors associated with multidrug resistance respectively. From the univariate analysis, we found that the odds of infection by MDROs were 1.33 times higher in females than males (COR 1.33; 95% CI 1.05–1.69; P-value 0.018). Patients of the under 5 age group (COR 2.35; 95% CI 1.06–5.37; P-value 0.037) and those who were 60 and more years of age (COR 1.41; 95% CI 1.05–1.89; P-value 0.023) had 2.35 times and 1.41 times more odds of MDROs infections respectively compared to those aged from 25 to 44 years. Hospitalized patients were 1.96 times more likely to have been infected by multidrug resistant bacteria than those who were not (COR 1.96; 95% CI 1.43–2.71; P-value < 0.001).

**Table 5:** Univariate logistic regression of factors associated with multidrug resistance.

Characteristic	COR^1,2^	95% CI^2^	p-value
**Sex**			
Male	–	–	
Female	1.33*	1.05, 1.69	0.018
**Age group**			
25–44	–	–	
Under 5	2.35*	1.06, 5.37	0.037
5–14	0.86	0.45, 1.60	0.600
15–24	1.55	0.98, 2.43	0.057
45–59	1.11	0.79, 1.56	0.500
60 +	1.41*	1.05, 1.89	0.023
**Hospitalization status**			
Outpatient	–	–	
Inpatient	1.96***	1.43, 2.71	< 0.001
**Isolated bacteria**			
*Klebsiella* spp*.*	–	–	
*Moraxella* spp*.*	0.35***	0.24, 0.51	< 0.001
*Staphylococcus* spp*.*	1.43	0.87, 2.39	0.200
*Acinetobacter* spp*.*	0.90	0.48, 1.68	0.700
*Enterobacter* spp*.*	1.11	0.64, 1.93	0.700
*Escherichia* spp*.*	1.32	0.92, 1.92	0.130
*Pseudomonas* spp*.*	0.24***	0.15, 0.38	< 0.001
Others	0.90	0.50, 1.59	0.700

^1^*p < 0.05; **p < 0.01; ***p < 0.001; ^2^*COR* crude odds ratio, *CI* confidence interval.

**Table 6:** Multivariable logistic regression of factors associated with multidrug resistance.

Characteristic	AOR^1,2^	95% CI^2^	p-value
**Hospitalization status**			
Outpatient	–	–	
Inpatient	1.78***	1.28, 2.49	< 0.001
**Isolated bacteria**			
*Klebsiella* spp*.*	–	–	
*Moraxella* spp*.*	0.38***	0.26, 0.54	< 0.001
*Staphylococcus* spp*.*	1.40	0.85, 2.34	0.200
*Acinetobacter* spp*.*	0.86	0.46, 1.60	0.600
*Enterobacter* spp*.*	1.15	0.66, 2.00	0.600
*Escherichia* spp*.*	1.32	0.91, 1.91	0.150
*Pseudomonas* spp*.*	0.23***	0.14, 0.37	< 0.001
Others	0.89	0.50, 1.59	0.700

^1^*p < 0.05; **p < 0.01; ***p < 0.001; ^2^*AOR* adjusted odds ratio, *CI* confidence interval.

The multivariable logistic regression revealed that only the inpatient status was positively associated with multidrug resistance. Generally, it increased by 1.78 times the odds of infections by MDROs in comparison to non-hospitalized patients (AOR 1.78; 95% CI 1.28–2.49; P-value < 0.001). There were also other factors such as infections by *Moraxella* spp*.* and *Pseudomonas* spp*.* which were found to lower the odds of multidrug resistance with statistical significance in both univariate and multivariable regression analyses.

## Discussion

Diagnostic stewardship is essential for health facilities at the local level. It contributes to the promotion of timely and adequate laboratory diagnostic testing. This involves the collection of samples, the identification of disease causing agents and the accurate as well as prompt reporting of results to guide the treatment of patients^21^. This is lacking in many hospitals in sub-Saharan Africa, with little or no monitoring of whether or not a newly admitted patient is a carrier of multidrug resistant germs, whose infections frequently lead to increased morbidity and mortality^22,23^. The present study had to fill the knowledge gap on the organisms involved in bacterial infections, their resistance profile and the prevalence of multidrug resistance as well as the associated risk factors in Northern Ghana.

Analysis of the data from this study showed that *Klebsiella* spp*.* was the most prevalent bacteria and had shown a very high level of resistance to Amoxicillin and to other antibiotics such as Tetracycline, Trimethoprim/Sulfamethoxazole, Ceftriaxone, and Cefotaxime. A study to determine the geographic distribution of *Klebsiella* spp*.* strains in health facilities that serve as referral centers in the northern, central, and southern belts of Ghana reported similar results. More than 70.0% of resistance has been found against 3rd generation cephalosporins (ceftriaxone, cefotaxime, and ceftazidime) among *Klebsiella* spp*.* strains, with a greater resistance to ampicillin^24^. There have been more than 100 acquired resistance genes identified in *Klebsiella* strains conferring them the ability to resist the effect of multiple antibiotic classes, notably polymyxins, beta-lactams, aminoglycosides, quinolones, and tigecycline^25–27^ which could consequently explain these high resistance levels. The existence of resistance genes has been documented among *Klebsiella* spp. strains in different parts of Ghana^28–30^ and in other countries of the West African sub-region^31–37^. This demonstrates the need to conduct molecular epidemiology studies to assess the resistance genotypes of these circulating strains and their spatial distribution.

Among the five (5) most prevalent bacteria, three (3) of them (*Staphylococcus* spp., *Klebsiella* spp., and *Pseudomonas* spp.) had strains classified as pathogens of the ESKAPE group (*Enterococcus faecium*, *Staphylococcus aureus*, *Klebsiella pneumoniae*, *Acinetobacter baumannii*, *Pseudomonas aeruginosa,* and *Enterobacter* species)^38^. These strains are well known in clinical settings and have the ability to resist the bactericidal or bacteriostatic actions of many antimicrobial agents by developing resistance mechanisms either by gene or plasmid acquisition or by genetic mutations^39,40^. The World Health Organization (WHO) listed these ESKAPE pathogens as being part of the most critical group of bacteria that represent a particular hazard in healthcare facilities like hospitals and nursing homes, where they can lead to serious and frequently fatal diseases like pneumonia and bloodstream infections^41^. In our study, blood was the 5th most prevalent specimen and multiple studies have reported the involvement of ESKAPE pathogens in bloodstream infections where they showed high levels of resistance to the antibiotics when tested^42–46^. In a study conducted in the United States, these pathogens were even associated with higher costs ($5500 more) and mortality (2.10% absolute increase)^47^. This shows that it is of major importance for countries such as Ghana to engage in research focused on exploring traditional African pharmacopeia for new antimicrobial molecules to help in the development of new antibiotics that can effectively combat these pathogens to minimize undesirable consequences.

The most representative specimen in our study was sputum (68.0%). This differs from a study on multidrug resistant bacteria conducted in Komfo Anokye Teaching Hospital (KATH) in Kumasi^10^ where the most representative specimen was urine (47.0%). This difference may be due to geographical location of the studies in Ghana. Our study was conducted in the northern region (in northern Ghana) while the study conducted at KATH was carried out in the Ashanti region in the southern part of Ghana. This geographical difference may explain the differences in the types of infections to which people in these regions are prone.

High resistance levels have been found in our study against Cefoxitin (74.0%), Tetracycline (71.0%), Trimethoprim/Sulfamethoxazole (68.0%), Ceftriaxone (68.0%), Cefotaxime (62.0%), Chloramphenicol (54.8%) and Erythromycin (51.0%) in Northern Ghana. These results are similar to those reported from the Greater Accra region (proportions of resistant isolates ranged from 37.9% and up to 69.1%)^48^ but also in Uganda (cefotaxime (74.2%) and cefoxitin (92.1%))^49^. These high levels of resistance could be explained by the fact that these antibiotics are the most commonly prescribed in health care facilities^50^. In addition, these antibiotics are easily accessible in pharmacies and drugstores over the counter. Therefore, self-medication practiced by the population in Ghana^51,52^ could be a contributing factor. It has already been reported that at the referral hospital in the Northern Region (Tamale Teaching Hospital); there is a high proportion of antibiotic abuse coupled with a high prevalence of incomplete treatment, off-label prescriptions, and potential interactions^17^. This may explain the particular case of these high proportions of resistance against the tested antibiotics in this study. This, therefore, demonstrates the urgent need for strengthening hospital-based antibiotic stewardship programs and extending its interventions to community levels. In sub-Saharan African countries, services for clinical bacteriology testing are usually reserved for higher levels of care and are therefore under-utilized^53^. As bacteriological diagnostics are performed in a very restrictive manner, this could result in patients with recurrent, difficult-to-treat and often resistant infections not receiving early and appropriate treatment because they have not had the opportunity to visit these institutions for accurate diagnosis and treatment. This leads to the selection of more resistant isolates and thus to high estimations of resistance levels, which may also explain the rates recorded in the present study.

A proportion of 41.6% was found for multidrug resistance of the strains isolated from TZPHRL. These results are lower than those found in northwest Nigeria (88.9%)^54^ and Ethiopia (85.8%)^15^ where significantly higher rates were reported. This difference could be explained by two factors. The first is related to the sample sizes used to determine the prevalence of MDR which were respectively 397 and 141 for Nigeria and Ethiopia while our study had a total of 1222. The second is that in their studies, they took into account only Gram-negative bacteria. However, the current study had both Gram-positive and Gram-negative bacteria. It is well known that Gram-negative bacteria (GNB) have a tendency to be more resistant to antimicrobial agents than Gram-positive bacteria^55^. This is due to the fact that their outer membrane gives them extra protection by preventing antibiotic molecules from penetrating the bacterial cell. In addition, GNBs are very high producers of extended-spectrum β-lactamases (ESBLs) and carbapenemases, which allows them to resist more antibiotics and thus express a greater multidrug resistance phenotype^56,57^. This could explain why the total prevalence of multidrug resistant strains is higher in these studies than in the current study. Magiorakos et al.^4^ reported various classes of antibiotics in their paper. However, some of these classes were not tested in our study. Thus, some bacteria that would normally be multidrug resistant would have been missed. This could also explain the relatively low proportion we found in our study compared to the previous studies.

About 28.00% of non-hospitalized patients were infected by multidrug resistant bacteria. This rate, although relatively low, it shows that these strains are circulating even in community settings where they could be transmitted either by Human-to-Human transmission or by the Human–Animal–Environment system. Typically, animal farming practices that employ excessive amounts of antibiotics can contaminate the agroecosystem through applying infected manure as fertilizer and irrigating crops with wastewater^58,59^. This is supported by findings from previous studies conducted in Northern Ghana^60,61^ and also in other parts of the country^62,63^ where resistance genes were detected in samples from animals, foods and the environment.

The current study found the female sex to be independently associated with multidrug resistance. These results corroborate those found in studies conducted in India and Saudi Arabia^64,65^. These results could be explained by the fact that women's exposure to antibiotic use is estimated to be 27.0%^66^ higher than men's. This is because they often use antibiotic treatment regimens in several phases of their lives, such as during pre-pregnancy, childbirth, and following abortion. In addition, young women, especially those who are sexually active, are at greater risk for vaginal infections, urinary tract infections, gonorrhea, and other diseases that may also lead to increased antibiotic prescriptions.

Out of the hospitalized patients from whom samples were taken for bacteriological diagnostics, 44.0% of the isolates were multidrug resistant and being hospitalized was also strongly associated with multidrug resistance in both univariate and multivariable analyses. This association has been found in previous investigations in other parts of the country^67,68^. This demonstrates that there is an urgent need to initiate interventions that will help to control this situation. An immediate action should be taken to reduce the circulation of MDROs in hospital settings by conducting observational studies on the practice of hygiene among health professionals working in hospitals within the study setting. In addition, there is the need to assess the state of knowledge regarding MDROs, their consequences and the means by which these strains can be transmitted in a health care facility. This allows for the creation of training content and adapt to the existing needs in the sector to effectively reinforce the capacities of healthcare professionals. Several studies have shown that capacity-building programs for healthcare professionals, particularly on the topic of hands hygiene, have been effective in controlling outbreaks of nosocomial infections involving multidrug resistant strains^69–71^.

### Strengths and limitations

A limitation of our study include the inability to capture other possible risk factors such as the length of stay in the health facility, previous antibiotic uptake and clinical history. Previous studies have reported them as risk factors associated with the infection by multidrug-resistant organisms in developed and developing countries^72–74^. However, due to the non-availability of such data, we were unable to investigate them in our context. In addition, due to the secondary nature of the data used in this study, we could not examine the prevalence of ESBLs and coagulase-negative *Staphylococcus* *aureus*.

This study has a number of strengths. Among others, we analyzed a large number of samples from at the TZPHRL, which is an integral part of Ghana health services and the public health system. This study also covers a 48-month period and therefore provides a snapshot of the situation in the Northern Region. This is because the samples were brought from different parts of the region.

In summary, we found high resistance rates against the tested antibiotics in the Northern region. Hospitalization was a risk factor for infections by multidrug resistant organisms. Thus, it is important to strengthen antibiotic stewardship programs while giving refresher training to healthcare professionals regarding topics such as infection control and prevention as well as diagnostic stewardship. Further studies using molecular epidemiology and mathematical modeling are also required to formulate recommendations for decision-makers at different levels of the health system in Ghana. This will contribute to strengthening the different initiatives that are taken to tackle the progression of antimicrobial resistance at the national level.

## Supplementary Information

Supplementary Table S1.

Supplementary Table S2.

Supplementary Table S3.

## Data Availability

The data underlining the conclusions drawn in this study are contained within the manuscript. However, the dataset can be made available upon reasonable request from the corresponding author.

## Abbreviations

TZPHRL: Tamale Zonal Public Health Reference Laboratory
COR: Crude odds ratio
AOR: Adjusted odds ratio
AMR: Antimicrobial resistance
MDROs: Multidrug-resistant organisms
CI: Confidence interval
TTH: Tamale Teaching Hospital
GNB: Gram-negative bacteria
ESBLs: Extended-spectrum β-lactamases
