# Supplementary material for: Bacterial infections epidemiology and factors associated with multidrug resistance in the northern region of Ghana
Source: Sci Rep. 2022 Dec 21;12:22069. doi: 10.1038/s41598-022-26547-7 (PMC9772187; doi:10.1038/s41598-022-26547-7)
Supplement: Supplementary file 1 — Supplementary Table S1. [file 41598_2022_26547_MOESM1_ESM.pdf]

Bacterial Infections Epidemiology and factors associated with Multidrug Resistance in the northern region of Ghana

Jean-Pierre Gnimatin[1*], Enoch Weyori[2], Shimea M. Agossou[3], & Martin Nyaaba Adokiya[4]

S1 Table. Samples received at the TZPHRL between June 2018 and May 2022

Sample	N = 1,222[1]
Sputum	827 (68%)
Urine	133 (11%)
High vaginal swab	78 (6.4%)
Wound Swab	74 (6.1%)
Blood	62 (5.1%)
Aspirates	18 (1.5%)
Gastric lavage	4 (0.3%)
Stool	4 (0.3%)
Pus	3 (0.2%)
Throat Swab	3 (0.2%)
Semen	2 (0.2%)
Tissue	2 (0.2%)
Urethral Swab	2 (0.2%)
Abdominal Abscess	1 (<0.1%)
Bone Scrab	1 (<0.1%)
Cerebrospinal Fluid	1 (<0.1%)
Nails	1 (<0.1%)
Scrotal Swab	1 (<0.1%)

[1] n (%)
