# Supplementary material for: Bacterial infections epidemiology and factors associated with multidrug resistance in the northern region of Ghana
Source: Sci Rep. 2022 Dec 21;12:22069. doi: 10.1038/s41598-022-26547-7 (PMC9772187; doi:10.1038/s41598-022-26547-7)
Supplement: Supplementary file 2 — Supplementary Table S2. [file 41598_2022_26547_MOESM2_ESM.pdf]

Bacterial Infections Epidemiology and factors associated with Multidrug Resistance in the northern region of Ghana

Jean-Pierre Gnimatin[1*], Enoch Weyori[2], Shimea M. Agossou[3], & Martin Nyaaba Adokiya[4]

S2 Table. Sex-based distribution of the isolated bacteria

Isolated bacteria	Female<br>N = 593[1]	Male<br>N = 590[1]	Overall<br>N = 1,183[1]	p-value[2]
				0.018
Klebsiella spp.	162 (51%)	158 (49%)	320 (100%)	
Moraxella spp.	115 (44%)	146 (56%)	261 (100%)	
Escherichia spp.	120 (62%)	75 (38%)	195 (100%)	
Pseudomonas spp.	62 (41%)	90 (59%)	152 (100%)	
Staphylococcus spp.	49 (56%)	39 (44%)	88 (100%)	
Enterobacter spp.	33 (53%)	29 (47%)	62 (100%)	
Acinetobacter spp.	22 (46%)	26 (54%)	48 (100%)	
Proteus spp.	5 (38%)	8 (62%)	13 (100%)	
Raoultella spp.	7 (64%)	4 (36%)	11 (100%)	
Streptococcus spp.	8 (89%)	1 (11%)	9 (100%)	
Salmonella spp.	2 (40%)	3 (60%)	5 (100%)	
Serratia spp.	1 (25%)	3 (75%)	4 (100%)	
Corynebacterium spp.	2 (67%)	1 (33%)	3 (100%)	
Citrobacter spp.	0 (0%)	2 (100%)	2 (100%)	
Enterococcus spp.	1 (50%)	1 (50%)	2 (100%)	
Micrococcus spp.	1 (50%)	1 (50%)	2 (100%)	
Pantoea spp.	1 (50%)	1 (50%)	2 (100%)	
Gardnerella spp.	1 (100%)	0 (0%)	1 (100%)	
Photobacterium spp.	0 (0%)	1 (100%)	1 (100%)	
Providencia spp.	0 (0%)	1 (100%)	1 (100%)	

**Isolated bacteria**	**Female** N = 593[1]	**Male** N = 590[1]	**Overall** N = 1,183[1]	**p-value**[2]
Sphingomonas spp.	1 (100%)	0 (0%)	1 (100%)	

[1] n (%); [2] Pearson's Chi-squared test
