# Supplementary material for: Bacterial infections epidemiology and factors associated with multidrug resistance in the northern region of Ghana
Source: Sci Rep. 2022 Dec 21;12:22069. doi: 10.1038/s41598-022-26547-7 (PMC9772187; doi:10.1038/s41598-022-26547-7)
Supplement: Supplementary file 3 — Supplementary Table S3. [file 41598_2022_26547_MOESM3_ESM.pdf]

**Bacterial Infections Epidemiology and factors associated with Multidrug Resistance in the northern region of Ghana**

Jean-Pierre Gnimatin[1*], Enoch Weyori[2], Shimea M. Agossou[3], & Martin Nyaaba Adokiya[4]

**S3 Table.** Distribution of the isolated bacteria by patient age group

| **Characteristic** | **Under 5** N = 27[1] | **5 – 14** N = 49[1] | **15 – 24** N = 97[1] | **25 – 44** N = 442[1] | **45 – 59** N = 211[1] | **60 +** N = 319[1] | **p-value**[2] |
|---|---|---|---|---|---|---|---|
| Bacteria | | | | | | | <0.001 |
| *Klebsiella spp.* | 3 (11%) | 7 (14%) | 24 (25%) | 126 (29%) | 54 (26%) | 96 (30%) | |
| *Moraxella spp.* | 4 (15%) | 11 (22%) | 11 (11%) | 104 (24%) | 55 (26%) | 72 (23%) | |
| *Escherichia spp.* | 0 (0%) | 7 (14%) | 28 (29%) | 73 (17%) | 33 (16%) | 47 (15%) | |
| *Pseudomonas spp.* | 2 (7.4%) | 4 (8.2%) | 8 (8.2%) | 53 (12%) | 29 (14%) | 49 (15%) | |
| *Staphylococcus spp.* | 13 (48%) | 5 (10%) | 9 (9.3%) | 32 (7.2%) | 9 (4.3%) | 11 (3.4%) | |
| *Enterobacter spp.* | 0 (0%) | 3 (6.1%) | 8 (8.2%) | 17 (3.8%) | 12 (5.7%) | 23 (7.2%) | |
| *Acinetobacter spp.* | 2 (7.4%) | 5 (10%) | 4 (4.1%) | 14 (3.2%) | 8 (3.8%) | 14 (4.4%) | |
| *Proteus spp.* | 0 (0%) | 2 (4.1%) | 0 (0%) | 5 (1.1%) | 6 (2.8%) | 1 (0.3%) | |
| *Raoultella spp.* | 0 (0%) | 0 (0%) | 2 (2.1%) | 5 (1.1%) | 1 (0.5%) | 3 (0.9%) | |
| *Streptococcus spp.* | 1 (3.7%) | 0 (0%) | 1 (1.0%) | 5 (1.1%) | 2 (0.9%) | 0 (0%) | |
| *Salmonella spp.* | 2 (7.4%) | 2 (4.1%) | 0 (0%) | 0 (0%) | 0 (0%) | 1 (0.3%) | |
| *Corynebacterium spp.* | 0 (0%) | 1 (2.0%) | 0 (0%) | 2 (0.5%) | 0 (0%) | 0 (0%) | |
| *Serratia spp.* | 0 (0%) | 0 (0%) | 1 (1.0%) | 2 (0.5%) | 0 (0%) | 0 (0%) | |
| *Citrobacter spp.* | 0 (0%) | 0 (0%) | 0 (0%) | 0 (0%) | 1 (0.5%) | 1 (0.3%) | |
| *Micrococcus spp.* | 0 (0%) | 0 (0%) | 1 (1.0%) | 0 (0%) | 1 (0.5%) | 0 (0%) | |
| *Pantoea spp.* | 0 (0%) | 0 (0%) | 0 (0%) | 2 (0.5%) | 0 (0%) | 0 (0%) | |
| *Enterococcus spp.* | 0 (0%) | 0 (0%) | 0 (0%) | 0 (0%) | 0 (0%) | 1 (0.3%) | |
| *Gardnerella spp.* | 0 (0%) | 1 (2.0%) | 0 (0%) | 0 (0%) | 0 (0%) | 0 (0%) | |
| *Photobacterium spp.* | 0 (0%) | 0 (0%) | 0 (0%) | 1 (0.2%) | 0 (0%) | 0 (0%) | |
| *Providencia spp.* | 0 (0%) | 1 (2.0%) | 0 (0%) | 0 (0%) | 0 (0%) | 0 (0%) | |

| Characteristic | Under 5<br>N = 27[1] | 5 – 14<br>N = 49[1] | 15 – 24<br>N = 97[1] | 25 – 44<br>N = 442[1] | 45 – 59<br>N = 211[1] | 60 +<br>N = 319[1] | p-value[2] |
|---|---|---|---|---|---|---|---|
| *Sphingomonas spp.* | 0 (0%) | 0 (0%) | 0 (0%) | 1 (0.2%) | 0 (0%) | 0 (0%) | |

[1] n (%), [2] Pearson's Chi-squared test
